# Retracted: Overexpression of NTRK1 Promotes Differentiation of Neural Stem Cells into Cholinergic Neurons

**DOI:** 10.1155/2019/3530959

**Published:** 2019-03-03

**Authors:** BioMed Research International

At the request of the authors and editors, the article titled “Overexpression of NTRK1 Promotes Differentiation of Neural Stem Cells into Cholinergic Neurons” [1] has been retracted. Based on concerns raised on PubPeer, the article was found to contain signs of image manipulation in Figure 2, where Figures 2(c) and 2(d) appear to show the same groups of cells although the two panels should show different experiments. Figure 2(c) appears to be a horizontally flipped and edited version of Figure 2(d), in which many image elements have been removed and several others have been moved and/or rotated/flipped.

The corresponding author apologizes to the readers and editors of BioMed Research International.
